# Optimization and Comparison of Synthetic Procedures for a Group of Triazinyl-Substituted Benzene-Sulfonamide Conjugates with Amino Acids

**DOI:** 10.3390/molecules22091533

**Published:** 2017-09-13

**Authors:** Dominika Krajčiová, Daniel Pecher, Vladimír Garaj, Peter Mikuš

**Affiliations:** 1 Department of Pharmaceutical Analysis and Nuclear Pharmacy, Faculty of Pharmacy, Comenius University in Bratislava, Odbojárov 10, SK-832 32 Bratislava, Slovakia; krajciova.dominika@fpharm.uniba.sk (D.K.); pecher1@uniba.sk (D.P.); 2 Toxicological and Antidoping Center, Faculty of Pharmacy, Comenius University in Bratislava, Odbojárov 10, SK-832 32 Bratislava, Slovakia; 3 Department of Pharmaceutical Chemistry, Faculty of Pharmacy, Comenius University in Bratislava, Odbojárov 10, SK-832 32 Bratislava, Slovakia; garajv@gmail.com

**Keywords:** 1,3,5-triazine conjugates, sulfonamides, amino acids, carboanhydrase inhibitors, synthesis optimization

## Abstract

Sulfonamides incorporating 1,3,5-triazine moieties can selectively and potently inhibit carbonic anhydrase transmembrane isoforms IX, XII, and XIV over cytosolic isoforms I and II. In the present work, a highly effective synthetic procedure was proposed for this group of potent cancerostatic drugs and compared with previously used methods. The synthesis of triazinyl-substituted benzene-sulfonamide conjugates with amino acids can be easily carried out using sodium carbonate-based water solution as a synthetic medium instead of *N,N*-Diisopropylethylamine/Dimethylformamide. The benefits of this synthetic procedure include: (i) high selectivity of the creation of disubstituted conjugates; (ii) several times higher yield (≥95%) than that achieved previously; (iii) elimination of organic solvents by the use of an environmental friendly water medium (green chemistry); (iv) simple and fast isolation of the product. The synthesis and resulting products were evaluated using TLC, IR, NMR, and MS methods. The present work demonstrates a significant advantage in providing shortened routes to target structures.

## 1. Introduction

Carbonic anhydrase (CA) IX and XII, as secondary tumor-related CAs, are found to be overexpressed in cervical, breast, bladder, and non-small cell lung cancers by instigating hypoxia and causing acidification in the extracellular region, which leads to metastatic spread of these tumor cells. Furthermore, the acidification can render classical cancer treatments ineffective, particularly those utilizing basic antitumor drugs and radiotherapy. It is therefore suggested that the inhibition of CA IX/CA XII could be an attractive alternative option for anticancer therapy [1,2,3].

Recently, it was shown that sulfonamides incorporating 1,3,5-triazine moieties can selectively and potently inhibit carbonic anhydrase transmembrane isoforms IX, XII, and XIV over cytosolic isoforms I and II [4,5,6,7,8]. The biological activity as well as the water solubility of the triazinyl-substituted benzene-sulfonamides were changed via the modification of the chemical structure after introducing various moieties, namely aliphatic amines, methyl esters of amino acids, amino acids and amino acid derivatives, ammonia, hydrazine, primary and secondary amines, amino-sulfon amides, alcohols, phenols, anilines, morpholine, tert-butylamine, *N*-methyl-2-amino ethanol and 1,3-diaminopropane, water, methylamine, or aliphatic alcohols (methanol and ethanol). Here, a reaction of triazinyl-substituted benzene-sulfonamide with amino acids afforded a new series of highly potent conjugates with enhanced polarity [4,5]. The reaction was carried out in inert atmosphere (N_2_) at 60 °C in dry DMF (Dimethylformamide) and DIPEA (*N,N*-Diisopropylethylamine). The reaction was quenched with slush, and the precipitate formed was collected by filtration, washed with H_2_O, and dried under high vacuum to give the required compound as a white solid. Declared yields for amino acid (glycine, β-alanine) conjugates ranged in the interval of 32–55%. These parameters indicate that there is a need to revise the established synthetic procedure with respect to the reaction yield and working conditions.

An aim of the present work was to propose a more effective synthetic procedure for the preparation of triazinyl-substituted benzene-sulfonamide conjugates with amino acids. Current criteria for such synthesis include: (i) a significant increase in the reaction yield; (ii) the minimization of organic solvents employed; and (iii) the simplification of the synthetic and isolation procedure. As model amino acids, glycine and alanine were selected to investigate and compare various synthetic strategies, including the proposed strategies and those previously employed (based on the DIPEA/DMF procedure).

## 2. Results and Discussion

**Scheme 1:**
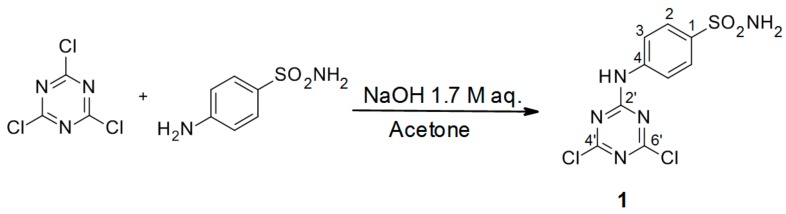
Synthesis of 4-(4′,6′-dichloro-1′,3′,5′-triazin-2′-ylamino)-benzene-sulfonamide precursor **1.** Reagents and conditions: 4-aminobenzenesulfanilamide, cyanuric chloride, acetone, at 0 °C, was added to an aqueous solution of NaOH, stirred for 1 h, and the reaction was subsequently quenched by the addition of slush (100 mL), after which the solid was filtered off. The scheme is reprinted from [4], *with permission of the publisher*. Product yield: 95%.

**Scheme 2:**
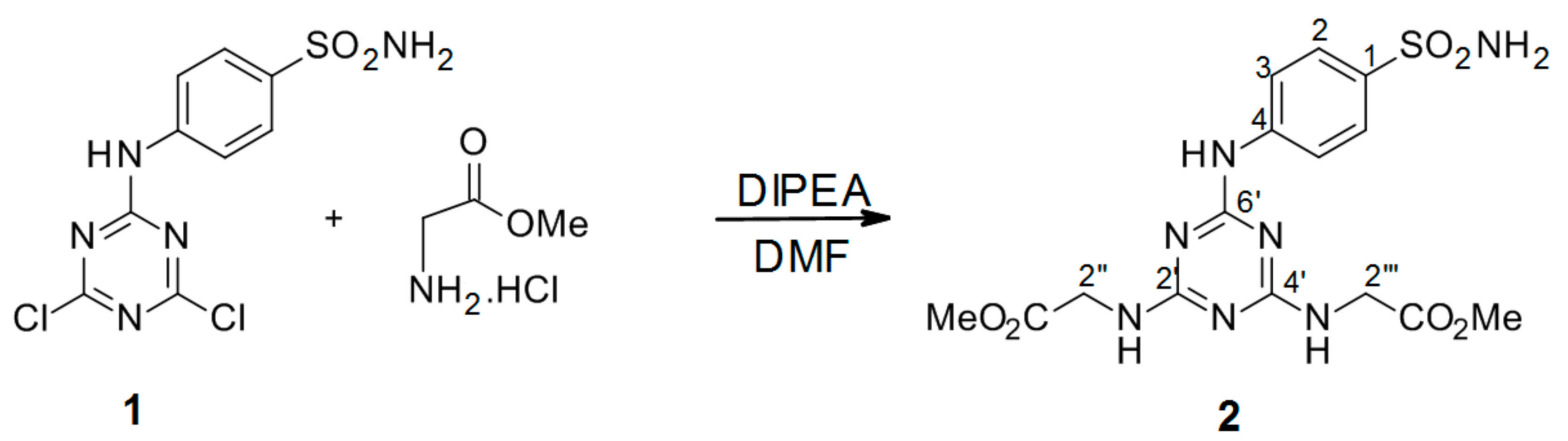
Synthesis of dimethyl 2′′,2′′′-[6′-(4-sulfamoylphenylamino)-1′,3′,5′-triazine-2′,4′-diyl]-bis(azanediyl)diacetate intermediate **2.** Reagents and conditions: a mixture of methyl 2-aminoacetate hydrochloride, DMF, and DIPEA was stirred for 2 h at r.t. Then, 4-(4′,6′-dichloro-1′,3′,5′-triazin-2′-ylamino)-benzene-sulfonamide **1** dissolved DMF was added, and the mixture was stirred at 90 °C under an argon atmosphere overnight. Product yield: 37%.

**Scheme 3:**
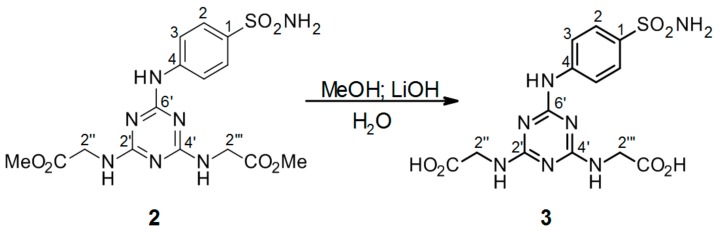
Hydrolysis of 2′′,2′′′-[6′-(4-sulfamoylphenylamino)-1′,3′,5′-triazine-2′,4′-diyl]-bis(azanediyl)diacetate intermediate **2** to product **3.** Reagents and conditions: a mixture of 2′′,2′′′-[6′-(4-sulfamoylphenylamino)-1′,3′,5′-triazine-2′,4′-diyl]-bis(azanediyl)diacetate **2**, MeOH, LiOH, and H_2_O was stirred at r.t. for 3 h. Product yield: 64%.

**Scheme 4:**
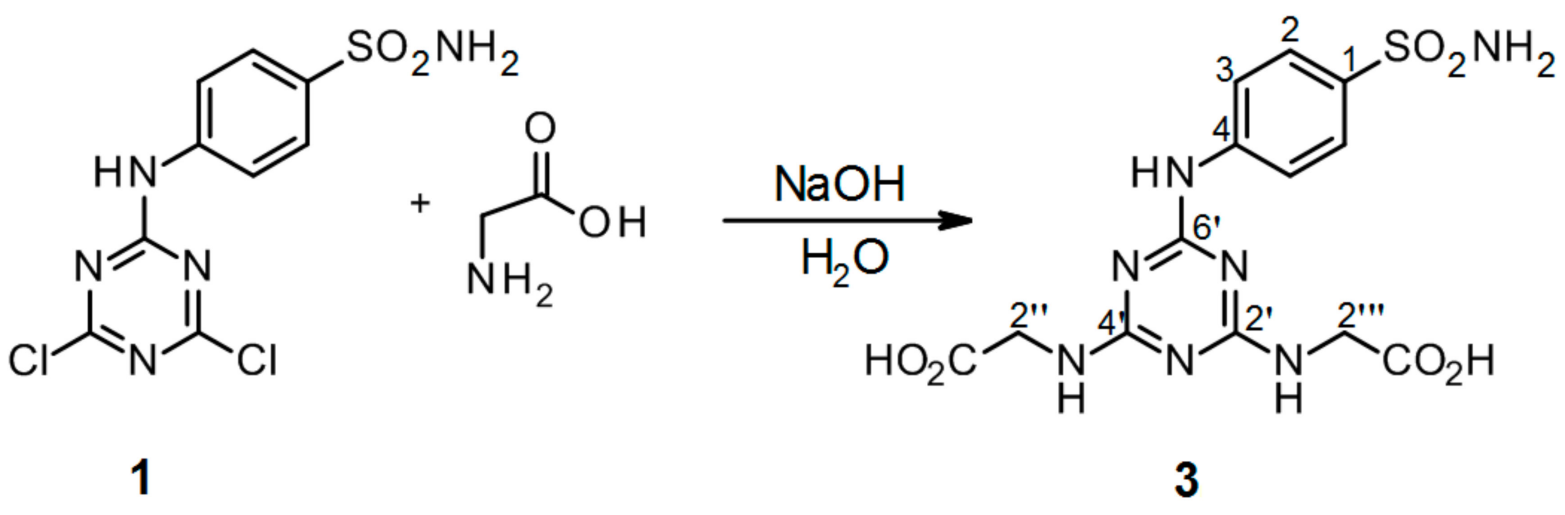
Synthesis of 2′′,2′′′-[6′-(4-sulfamoylphenylamino)-1′,3′,5′-triazine-2′,4′-diyl]-bis(azanediyl)diacetic acid **3.** Reagents and conditions: 4-(4′,6′-dichloro-1′,3′,5′-triazin-2′-ylamino)-benzene-sulfonamide **1,** glycine, NaOH, and H_2_O were refluxed for 24 h. Product yield: 65%.

**Scheme 5:**
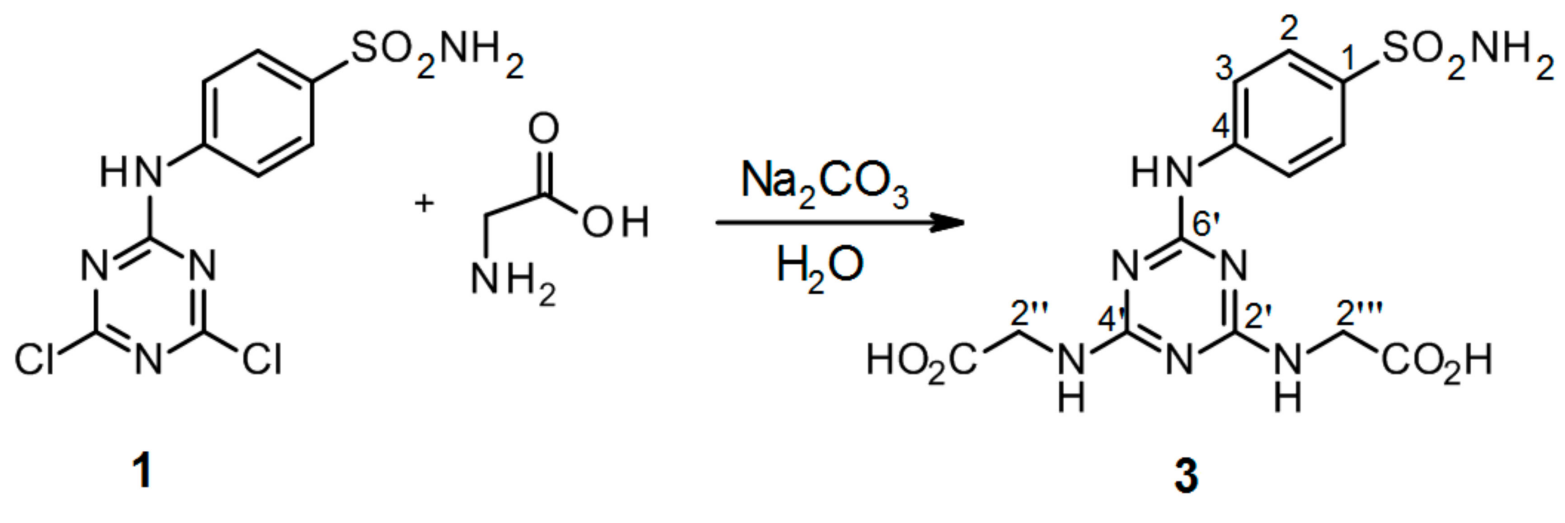
Synthesis of 2′′,2′′′-[6′-(4-sulfamoylphenylamino)-1′,3′,5′-triazine-2′,4′-diyl]-bis(azanediyl)diacetic acid **3**. Reagents and conditions: 4-(4′,6′-dichloro-1′,3′,5′-triazin-2′-ylamino)-benzene-sulfonamide **1,** glycine, Na_2_CO_3_, and H_2_O were refluxed for 24 h. Product yield: 96%.

**Scheme 6:**
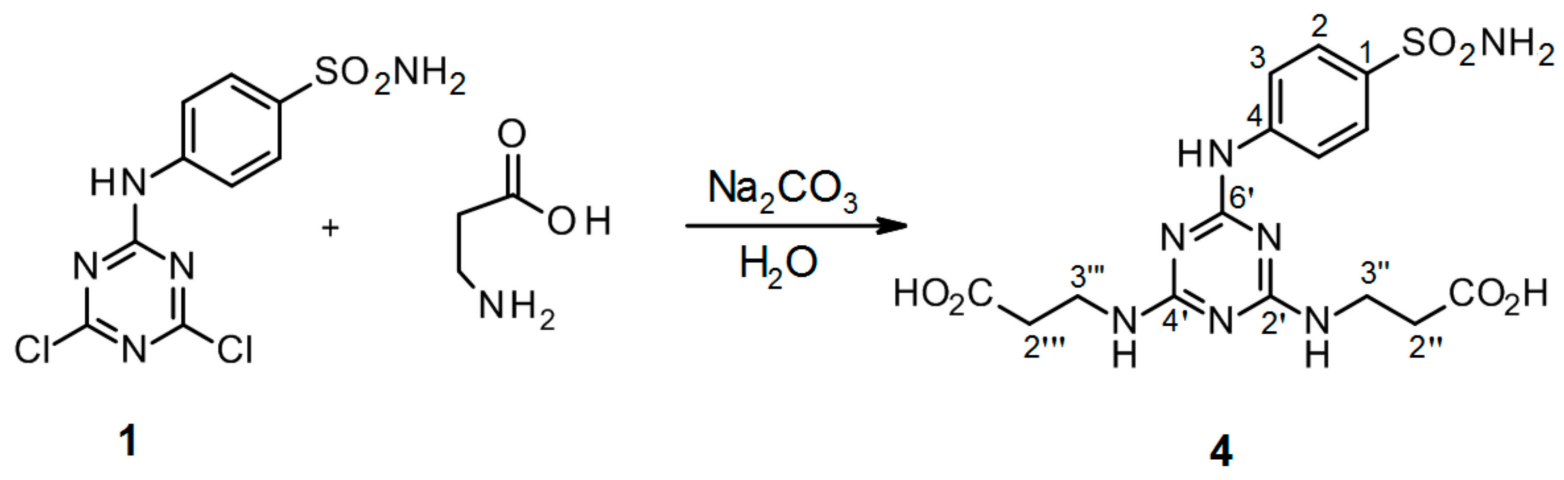
Synthesis of 3′′,3′′′-[6′-(4-sulfamoylphenylamino)-1′,3′,5′-triazine-2′,4′-diyl]-bis(azanediyl)dipropanoic acid **4**. Reagents and conditions: 4-(4′,6′-dichloro-1′,3′,5′-triazin-2′-ylamino)-benzene-sulfonamide **1**, β-alanine, Na_2_CO_3_, and H_2_O were refluxed for 24 h. Product yield: 95%.

We studied the possibilities of effectivization of the synthetic procedure for the preparation of triazinyl-substituted benzene-sulfonamide conjugates with amino acids. Two previously used amino acids, i.e., glycine and β-alanine, were selected in the present work to investigate and compare various synthetic strategies with the established one based on a 4-(4′,6′-dichloro-1′,3′,5′-triazin-2′-ylamino)-benzene-sulfonamide precursor **1** (Scheme 1) and a DIPEA/DMF environment [4]. The intermediates and products were synthesized via Scheme 2, Scheme 3, Scheme 4, Scheme 5 and Scheme 6, isolated, purified, and identified (see corresponding NMR, IR, and HPLC-UV graphical profiles S1-S14 in the Supplementary file).

Firstly, we investigated the synthesis of the amino acid conjugates prepared in the DIPEA/DMF environment through esters of amino acids and the subsequent hydrolysis of the substituted products. The representative synthesis of dimethyl 2′′,2′′′-[6′-(4-sulfamoylphenylamino)-1′,3′,5′-triazine-2′,4′-diyl]-bis(azanediyl)diacetate intermediate **2** is shown in Scheme 2, while the hydrolysis of the intermediate **2** is shown in Scheme 3. Although the yield of the intermediate **2** was 37%, the yield of the final product **3** from this was only 64%. This means that the real yield of the final product **3** (prepared according to Scheme 2 and Scheme 3) was ca. 24%, which was less than the 32% value declared for 2′′,2′′′-[6′-(4-sulfamoylphenylamino)-1′,3′,5′-triazine-2′,4′-diyl]-bis(azanediyl)diacetic acid conjugate in Reference [4].

Secondly, a water environment (containing NaOH) instead of an organic one was tested in order to enhance the yield of the product and its isolation. The representative synthesis of conjugate **3** is shown in Scheme 4. Although the expected product **3** was obtained in a reasonable amount in solid state (ca. 81% of the theoretical yield), applied NMR as well as HPLC-UV/MS methods indicated some impurities in the sample. HPLC-UV/MS detected a major amount (75.1%/53.9%) of **3** with two substituted glycines (*m*/*z* 398.0877), and minor amounts of impurities related to **3** containing one substituted glycine and one substituted OH (*m*/*z* 341.0663) (11.0%/9.5%), or two substituted OH groups instead of glycine (*m*/*z* 284.0448) (2.7%/36.6%). According to the quantitative NMR, the amount of pure product **3** from the reaction mixture was ca. 65%. This is a considerably higher yield compared to that declared in the literature for 2′′,2′′′-[6′-(4-sulfamoylphenylamino)-1′,3′,5′-triazine-2′,4′-diyl]-bis(azanediyl)diacetic acid [4]. However, there is a need for an additional sample preparation to isolate product **3** (prepared according to Scheme 4) from its minor structurally related impurities.

Thirdly, a water environment replacing NaOH with Na_2_CO_3_ was tested in order to prevent the creation of minor structurally related impurities (mainly hydroxy-derivatives), and further enhance the yield of the product and improve its isolation. The representative synthesis of conjugate **3** is shown in Scheme 5. Here, the expected product **3** was obtained in very high amounts in the solid state (96% of the theoretical yield). Applied NMR as well as HPLC-UV/MS methods did not indicate any significant impurities in the sample, so no further purification procedure was necessary. Theoretical and experimental yields were in good agreement, and the HPLC-UV analysis indicated 95.51% purity of the product **3** (see Figure S13 in the Supplementary material). Relative error between the theoretical and HPLC-UV values was 0.51%. According to these findings, the real yield of pure product **3** (prepared according to Scheme 5) from the reaction mixture was higher than 95%. It was the highest yield in comparison with those obtained for 2′′,2′′′-[6′-(4-sulfamoylphenylamino)-1′,3′,5′-triazine-2′,4′-diyl]-bis(azanediyl)diacetic acid **3** by any other procedure. The compared procedures included (i) those performed in our work (see Scheme 2, Scheme 3 and Scheme 4) ranging in 24–65% yields as well as (ii) those published in the literature [4] with the yield value of 32%.

This remarkable improvement in the yield of triazinyl-substituted benzene-sulfonamide conjugates with amino acids was demonstrated with the glycine conjugate **3** (prepared according to Scheme 5) in addition to β-alanine conjugate **4** (prepared according to Scheme 6). Theoretical and experimental yields were in good mutual agreement. The HPLC-UV analysis indicated 94.98% purity of the product **4** (see Figure S14 in the Supplementary Material) while the theoretical value was 98%. Relative error between the theoretical and HPLC-UV values was 3.08%. Hence, it was confirmed that the synthetic strategy based on a water environment with Na_2_CO_3_ provided at least 95% yield of 3′′,3′′′-[6′-(4-sulfamoylphenylamino)-1′,3′,5′-triazine-2′,4′-diyl]bis(azanediyl)dipropanoic acid **4**. This yield is considerably higher than the value of 55% published in the literature [4].

## 3. Experimental Protocols

### 3.1. Chemistry

All reagents were purchased from Aldrich (St. Louis, MO, USA) and AppliChem GmbH (Darmstadt, Germany) and were used without further purification. The reaction was monitored by thin-layer chromatography carried out on silica gel plates (60 F_254_, MERCK, Darmstadt, Germany) as the stationary phase; ethylacetate/*n*-hexane or MeOH/DCM were used as eluents. Spots were visualized under UV light (245 nm). Nuclear magnetic resonance (^1^H-NMR, ^13^C-NMR) spectra were recorded using a Varian MERCURY plus 300 MHz spectrometer (Varian, Palo Alto, CA, USA) in DMSO-*d*_6_ at r.t. and at 80 °C. Chemical shifts were reported in parts per million (ppm, d) downfield from TMS (Tetramethylsilane). Splitting patterns are described as singlet (s), doublet (d), triplet (t), quartet (q), multiple (m), broad (br), and doublet of doublet (dd). Infrared (IR) spectra were performed on Perkin-Elmer UATR Two (PerkinElmer Ltd., Beaconsfield, UK) and were recorded as KBr plates. The absorption bands (ν_max_) are given in wave numbers (cm^−1^) and the signal strength is given as weak (w), strong (s), or medium (m). All of the HPLC-UV/MS analyses were performed on a chromatographic apparatus consisting of an LC Agilent Infinity System (Agilent Technologies, Santa Clara, CA, USA) equipped with a gradient pump (1290 Bin Pump VL), an automatic injector (1260 HiPals), and a column thermostat (1290 TCC). The LC system was coupled with a photodiode array detector (Infinity 1290 DAD) and a quadrupole time-of-flight mass spectrometer (6520 Accurate Mass Q-TOF LC/MS). Q-TOF was equipped with an electrospray ionization source operated in positive ionization mode. For data acquisition and processing, a computer with Mass Hunter software(version MassHunter Workstation B 04.00, Agilent Technologies, Santa Clara, CA, USA) was used. The HPLC-HRMS analysis was carried out using HILIC column—SeQuant^®^ ZIC^®^-HILIC, 2.1 × 100 mm, 3.5 µm obtained from Merck KGaA, Darmstadt, Germany. The mobile phases consisted of acetonitrile and 5 mM solution of ammonium acetate pH 5.8 (adjusted with acetic acid). The flow rate of the mobile phase was set at 400 µL/min and 5.0 µL of the sample were injected into the column. The overall time of analysis was 24 min. Gradient elution was used with the column oven temperature set at 35 °C. The following MS parameters were used for all measurements: drying gas temperature 325 °C, drying gas flow 10 L.min^-1^, nebulizing gas pressure 40 psi, ESI source voltage 3500 V, fragmentor voltage 140 V, collision gas N_2_.

### 3.2. General Procedure for the Synthesis of Compounds ***1–4***

#### 3.2.1. Synthesis of 4-(4′,6′-Dichloro-1′,3′,5′-triazin-2′-ylamino)-benzene-sulfonamide Precursor **1** (Scheme 1)

A 1.0 M solution of 4-aminobenzenesulfanilamide (17.2 g, 0.1 mol, 1.0 equiv) in acetone (100 mL) was added dropwise to a vigorously stirred suspension of cyanuric chloride (18.4 g, 1.0 equiv) in the same solvent (100 mL) at 0 °C. The white slurry was stirred at the same temperature for 30 min. Then, a 1.7 M aqueous solution of NaOH (4.0 g, 1.0 equiv) was added over a period of 20 min. Stirring was continued for 1 h, and the reaction was quenched by the addition of slush (100 mL), after which the solid was filtered off. Crystallization from acetone afforded the title compound **1** as a white solid [4].

*4-(4′,6′-Dichloro-1′,3′,5′-triazin-2′-ylamino)-benzene-sulfonamide* (**1**): The product was obtained as a white solid in 95% yield. IR (KBr): 3294 (s), 3211 (m), 2343 (w), 1621 (s), 1538 (s), 1494 (s), 1380 (m), 1328 (s), 1226 (s), 1164 (s), 1123 (m), 963 (w), 722 (s), 590 (m), 547 (s), 482 (m); ^1^H-NMR (DMSO-*d*_6_) δ: 11.43 (s, 1H, Ar-NH, Notice: The moiety was underlined to indicate the hydrogen to which the signal refers.), 7.86 (d, 2H, *J* =9.0 Hz, 2 × H-2), 7.79 (d, 2H, *J* = 9.0 Hz, 2 × H-3), 7.34 (s, 2H, SO_2_NH_2_); ^13^C-NMR (DMSO-*d*_6_) δ: 170.25, 169.47, 164.40, 140.44, 140.32, 127.11, 121.55. HRMS (ESI/QTOF) *m*/*z*: [M + H]^+^ Calcd. for [C_9_H_7_N_5_SO_2_C_l2_H]^+^ 319.9770; Found: 319.9772.

#### 3.2.2. Synthesis of Dimethyl 2′′,2′′′-[6′-(4-Sulfamoylphenylamino)-1′,3′,5′-triazine-2′,4′-diyl]-bis(azanediyl) Diacetate Intermediate **2** (Scheme 2)

*4-(4′,6′-Dichloro-1′,3′,5′-triazin-2′-ylamino)-benzene-sulfonamide* **1.** (0.2 g, 1.0 equiv) was dissolved in 2 mL dry DMF. The mixture of methyl 2-aminoacetate hydrochloride (4.5 equiv) in 2 mL DMF and DIPEA (9.0 equiv) was stirred for 2 h at r.t. After 2 h, a solution of 4-(4′,6′-dichloro-1′,3′,5′-triazin-2′-ylamino)-benzene-sulfonamide **1** was added dropwise and the reaction was stirred at 90 °C under an argon atmosphere. The reaction ran overnight and was monitored by TLC (Thin Layer Chromatography). The next day, the reaction was quenched with slush and the precipitate formed was collected by filtration, washed with H_2_O, and dried under high vacuum to give the title compound as a white solid.

*2′′,2′′′-[6′-(4-Sulfamoylphenylamino)-1′,3′,5′-triazine-2′,4′-diyl]-bis(azanediyl)diacetate* (**2**): The product was obtained as a white solid in 37% yield. IR (KBr): 3427 (m), 3367 (m), 3263 (m), 3005 (w), 2361 (w), 1734 (s), 1569 (s), 1500 (s), 1411 (s), 1317 (s), 1153 (s), 987 (m), 903 (w), 807 (s), 590 (m), 541 (m); ^1^H-NMR (DMSO-*d*_6_, 80°C) δ: 9.16 (s, 1H, Ar-NH), 7.86 (d, 2H, *J* = 8.8 Hz, H-2), 7.66 (d, 2H, *J* = 8.8 Hz, H-3), 7.07 (s, 2H, CH_2_-NH-), 6.95 (s, 2H, SO_2_NH_2_), 4.01 (d, 4H, *J* = 5.3 Hz, H-2′′, H-2′′′), 3.64 (s, 6H, 2 × CH_3_); ^13^C-NMR (DMSO-*d*_6_, 80 °C) δ: 171.27, 166.33, 164.50, 143.99, 137.00, 126.54, 119.35, 51.80, 42.65. HRMS (ESI/QTOF) *m*/*z*: [M + H]^+^ Calcd. for [C_15_H_19_N_7_SO_6_H]^+^ 426.1190; Found: 426.1190.

#### 3.2.3. Hydrolysis of Intermediate **2** (Scheme 2) to Product 2′′,2′′′-[6′-(4-Sulfamoylphenylamino)-1′,3′,5′-triazine-2′,4′-diyl]-bis(azanediyl)diacetic Acid **3** (Scheme 3)

*2′′,2′′′-[6′-(4-Sulfamoylphenylamino)-1′,3′,5′-triazine-2′,4′-diyl]-bis(azanediyl) diacetate* **2.** (0.10048 g) was dissolved in 4 mL MeOH. LiOH (4.0 eguiv) was dissolved in 4 mL H_2_O and added to the methanol solution of compound **2**. The mixture was stirred at r.t. and monitored by TLC. After 3 h, the hydrolysis was terminated (before the addition of 1M HCl, methanol was evaporated) by the addition of a few drops of 1M HCl until a white precipitate was no longer produced. The precipitated mixture was filtered and the precipitate was dried under high vacuum.

*2′′,2′′′-[6′-(4-Sulfamoylphenylamino)-1′,3′,5′-triazine-2′,4′-diyl]-bis(azanediyl)diacetic acid* **(3)**: The product was obtained as a white solid in 64% yield. IR (KBr): 3264 (w), 2361 (w), 1734 (w), 1591 (s), 1504 (s), 1406 (s), 1312 (m), 1229 (m), 1155 (s), 905 (w), 779 (m), 587 (m), 544 (m); ^1^H-NMR (DMSO-*d*_6_, 80 °C) δ: 9.29 (s, 1H, Ar-NH), 7.88 (d, 2H, *J* = 8.9 Hz, H-2), 7.68 (d, 2H, *J* = 8.9 Hz, H-3), 7.01 (br s, 4H, SO_2_NH_2_, 2 × CH_2_-NH), 3.96 (br s, 4H, 2 × CH_2_-NH); ^13^C-NMR (DMSO-*d*_6_, 80 °C) δ: 171.87, 165.72, 163.98, 143.83, 137.12, 126.65, 119.33, 42.70. HRMS (ESI/QTOF) *m*/*z*: [M + H]^+^ Calcd. for [C_13_H_15_N_7_SO_6_H]^+^ 398.0877; Found: 398.0877.

#### 3.2.4. Synthesis of 2′′,2′′′-[6′-(4-Sulfamoylphenylamino)-1′,3′,5′-triazine-2′,4′-diyl]-bis(azanediyl)diacetic Acid **3** (Scheme 4)

*4-(4′,6′-Dichloro-1′,3′,5′-triazin-2′-ylamino)-benzene-sulfonamide* **1.** (0.1 g, 1.0 equiv) was stirred in H_2_O (2 ml) at r.t. for 10 min. Then alkaline solution of glycine (2.4 equiv of Gly and 5.0 equiv of NaOH) was added dropwise into precursor **1**. The mixture was refluxed for 24 h and monitored by TLC. The synthesis was terminated by the addition of a few drops of 1M HCl until a white precipitate was no longer produced. The product was filtered off and dried under high vacuum.

*2′′,2′′′-[6′-(4-Sulfamoylphenylamino)-1′,3′,5′-triazine-2′,4′-diyl]-bis(azanediyl)diacetic acid* (**3**)**.** The product was obtained as a white solid in 81% yield. IR (KBr): 2968 (s), 2361 (s), 2343 (m), 1560 (m), 1498 (s), 1405 (s), 1308 (m), 1157 (s), 837 (w), 779 (m), 587 (m), 544 (s); ^1^H-NMR (DMSO-*d*_6_, 80 °C) δ: 9.14 (s, 1H, Ar-NH), 7.89 (d, 2H, *J* = 8.9 Hz, H-2), 7.67 (d, 2H, *J* = 8.9 Hz, H-3), 6.94 (s, 2H, SO_2_NH_2_), 6.88 (br s, 2H, 2 × CH_2_-NH), 3.94 (d, 4H, *J* = 6.2 Hz 2 × CH_2_-NH); ^13^C-NMR (DMSO-*d*_6_, 80 °C) δ: 172.06, 166.40, 164.50, 144.09, 136.83, 126.60, 119.28, 42.67. HRMS (ESI/QTOF) *m*/*z*: [M + H]^+^ Calcd. for [C_13_H_15_N_7_SO_6_H]^+^ 398.0877; Found: 398.0877 [C_13_H_15_N_7_SO_6_H]^+^; 341.0663 [C_11_H_12_N_6_SO_5_H]^+^; 284.0448 [C_9_H_9_N_5_SO_4_H]^+^.

#### 3.2.5. Synthesis of 2′′,2′′′-[6′-(4-Sulfamoylphenylamino)-1′,3′,5′-triazine-2′,4′-diyl]-bis(azanediyl)diacetic Acid 3 (Scheme 5)

*4-(4′,6′-Dichloro-1′,3′,5′-triazin-2′-ylamino)-benzene-sulfonamide* **1.** (0.2 g, 1.0 equiv) and glycine (3.0 equiv) were stirred in H_2_O (2 mL) at r.t. for 10 min. Then aqueous solution of Na_2_CO_3_ (4.0 equiv) was added dropwise into the mixture of precursor **1** and glycine. The mixture was refluxed for 24 h and monitored by TLC. The synthesis was terminated by the addition of a few drops of 1M HCl (up to pH = 2.0) until a white precipitate was no longer produced. The product was filtered off and dried under high vacuum.

*2′′,2′′′-[6′-(4-Sulfamoylphenylamino)-1′,3′,5′-triazine-2′,4′-diyl]-bis(azanediyl)diacetic acid* (**3**). The product was obtained as a white solid in 96% yield. IR (KBr): 2968 (w), 2360 (w), 2343 (w), 1683 (m), 1598 (m), 1507 (s), 1407 (s), 1159 (s), 1096 (w), 847 (w), 779 (s), 588 (m), 544 (s); ^1^H-NMR (DMSO-*d*_6_, 80 °C) δ: 9.17 (s, 1H, Ar-NH), 7.89 (d, 2H, *J* = 8.9 Hz, H-2), 7.67 (d, 2H, *J* = 8.9 Hz, H-3), 6.94 (br s, 4H, SO_2_NH_2_, 2 × CH_2_-NH), 3.95 (d, 4H, *J* = 5.2 Hz, 2 × CH_2_-NH); ^13^C-NMR (DMSO-*d*_6_, 80 °C) δ: 172.03, 166.23, 164.40, 144.04, 136.92, 126.66, 119.32, 42.70. HRMS (ESI/QTOF) *m*/*z*: [M + H]^+^ Calcd. for [C_13_H_15_N_7_SO_6_H]^+^ 398.0877; Found: 398.0878.

#### 3.2.6. Synthesis of 3′′,3′′′-[6′-(4-Sulfamoylphenylamino)-1′,3′,5′-triazine-2′,4′-diyl]bis(azanediyl) Dipropanoic acid **4** (Scheme 6)

*4-(4′,6′-Dichloro-1′,3′,5′-triazin-2′-ylamino)-benzene-sulfonamide* **1**. (0.2 g, 1.0 equiv) and β-alanine (3.0 equiv) were stirred in H_2_O (2 mL) at r.t. for 10 min. Then aqueous solution of Na_2_CO_3_ (4.0 equiv) was added dropwise into the mixture of precursor **1** and β-alanine. The mixture was refluxed for 24 h and monitored by TLC. The synthesis was terminated by the addition of a few drops of 1 M HCl (up to pH = 3.0) until a white precipitate was no longer produced. The product was filtered off and dried under high vacuum.

*3′′,3′′′-[6′-(4-Sulfamoylphenylamino)-1′,3′,5′-triazine-2′,4′-diyl]bis(azanediyl) dipropanoic acid* (**4**). The product was obtained as a white solid in 98% yield. IR (KBr): 2967 (m), 2361 (s), 2343 (m), 1630 (m), 1592 (s), 1509 (s), 1399 (m), 1163 (s), 889 (w), 678 (w), 581 (m), 552 (m); ^1^H-NMR (DMSO-*d*_6_, 80 °C) δ: 9.32 (br s, 1H, Ar-NH), 7.92 (d, 2H, *J* = 8.8 Hz, H-2), 7.69 (d, 2H, *J* = 8.8 Hz, H-3), 6.96 (br s, 4H, SO_2_NH_2_, 2 × CH_2_-NH), 3.53 (t, *J* = 7.1 Hz, 4H, H-2′′, H-2′′′), 2.54 (t, 4H, *J* = 7.1 Hz, H-3′′, H-3′′′); ^13^C-NMR (DMSO-*d*_6_, 80 °C) δ: 173.19, 164.70, 163.70, 143.83, 137.17, 126.68, 119.42, 36.98, 34.54. HRMS (ESI/QTOF) *m*/*z*: [M + H]^+^ Calcd. for [C_15_H_19_N_7_SO_6_H]^+^ 426.1190; Found 426.1190.

## 4. Conclusions

In this work, three synthetic procedures for a group of triazinyl-substituted benzene-sulfonamide conjugates with amino acids were designed and tested as alternatives to the already established procedure used for the preparation of these highly potent CA-inhibitors. It was clearly demonstrated that the synthetic procedure based on an amino acid (here glycine and β-alanine) attachment (i.e., nucleophilic substitution into two equivalent positions) to 4-(4′,6′-dichloro-1′,3′,5′-triazin-2′-ylamino)-benzene-sulfonamide in a water medium containing sodium carbonate provided significant improvement in terms of (i) the maximization of the reaction yield (≥95%, i.e., ca. 2–3-fold higher as that declared in the literature), (ii) the minimization of organic solvents employed (green synthesis), and (iii) the simplification of the synthetic and isolation procedure. The proposed procedure is also attractive for the effective preparation of required triazinyl-substituted benzene-sulfonamide conjugates with other amino acids containing an amino group as the only significant nucleophile in the structure. In this way, new analogues in the studied group of CA-inhibitors can be easily prepared.

## Supplementary Materials

Supplementary materials are available online.
