# Supplementary material for: Optimization and Comparison of Synthetic Procedures for a Group of Triazinyl-Substituted Benzene-Sulfonamide Conjugates with Amino Acids
Source: Molecules. 2017 Sep 13;22(9):1533. doi: 10.3390/molecules22091533 (PMC6151714; doi:10.3390/molecules22091533)
Supplement: Supplementary file 1 [file molecules-22-01533-s001.pdf]

# Supporting information for

# Optimization and Comparison of Synthetic Procedures for Group of Triazinyl-substituted Benzene-sulfonamide Conjugates with Amino Acids

**Dominika Krajčiová[1], Daniel Pecher[1, 2], Vladimír Garaj[3], Peter Mikuš[1, 2,*]**

[1] Department of Pharmaceutical Analysis and Nuclear Pharmacy, Faculty of Pharmacy, Comenius University in Bratislava, Odbojarov 10, SK-832 32 Bratislava, Slovak Republic; krajciova@fpharm.uniba.sk (D.K.); pecher1@uniba.sk (D.P.); mikus@fpharm.uniba.sk (P.M.)
[2] Toxicological and Antidoping Center, Faculty of Pharmacy, Comenius University in Bratislava, Odbojárov 10, SK-832 32 Bratislava, Slovak Republic
[3] Department of Pharmaceutical Chemistry, Faculty of Pharmacy, Comenius University in Bratislava, Odbojarov 10, SK-832 32 Bratislava, Slovak Republic; garaj@fpharm.uniba.sk (V.G.)

* Correspondence: mikus@fpharm.uniba.sk (P.M.); Tel.: +421-2-501-17-243 (P.M.)

## CONTENTS


| | | |
|---|---|---|
| Copies of NMR spectra | compounds **1**, **2, 3, 4** | **Figures S1-S6** |
| Copies of IR spectra | compounds **1**, **2, 3, 4** | **Figures S7-S12** |
| Copies of HPLC-UV chromatograms | compounds **3, 4** | **Figures S13 and S14** |

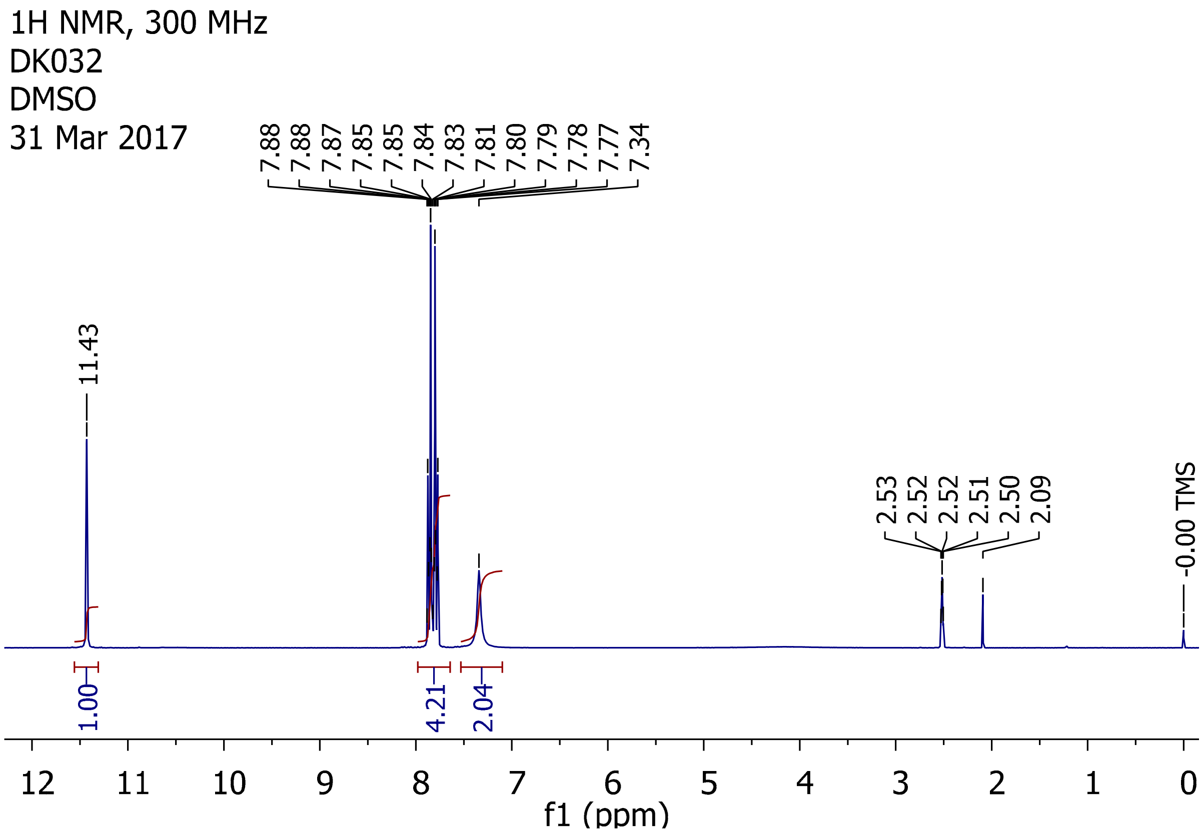


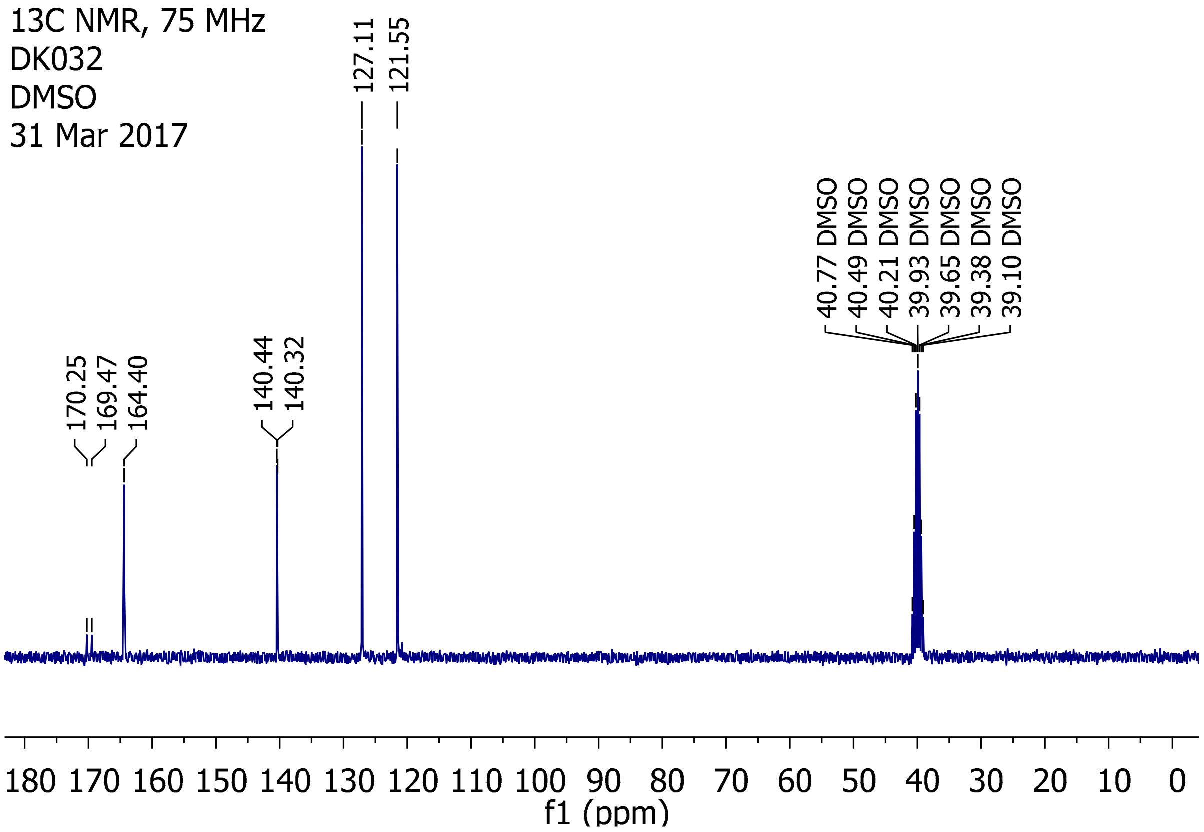


**Figure S1:** $^{1}$H and $^{13}$C NMR spectra of 4-(4',6'-dichloro-1',3',5'-triazin-2'-ylamino)-benzenesulfonamide **1**

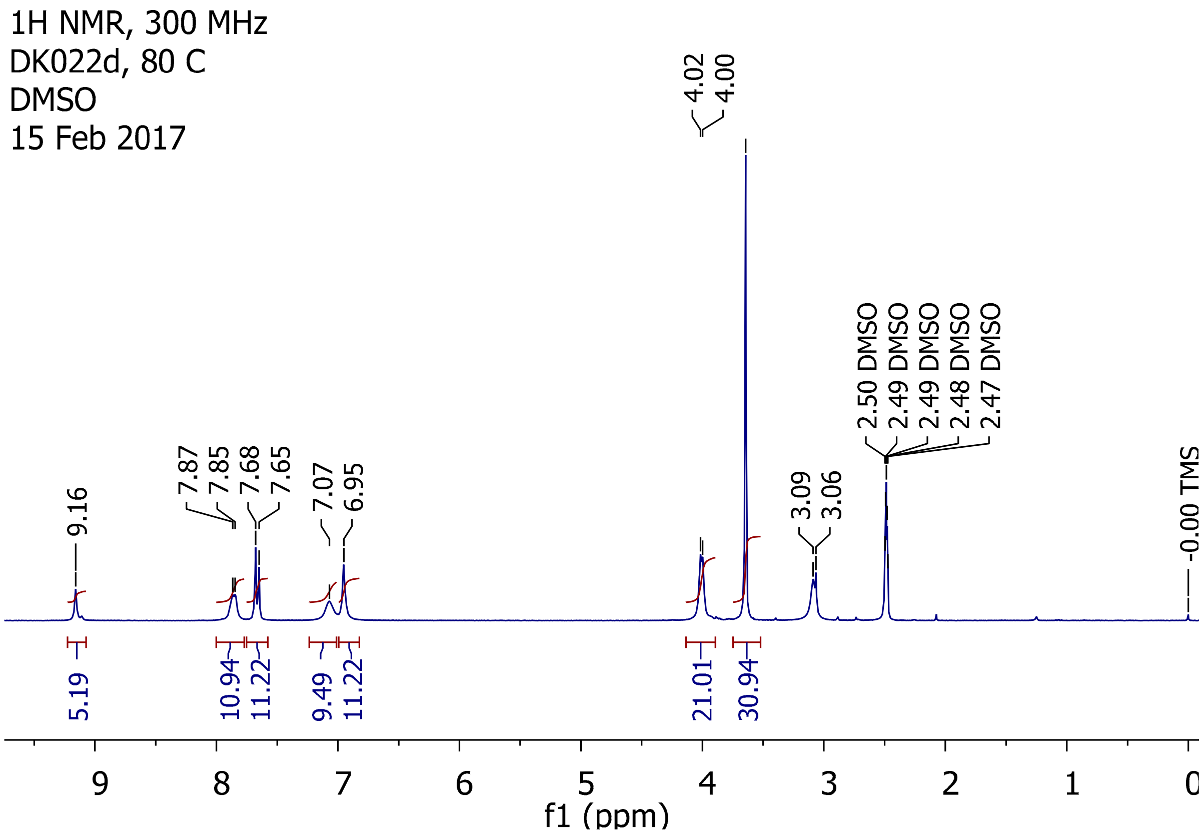


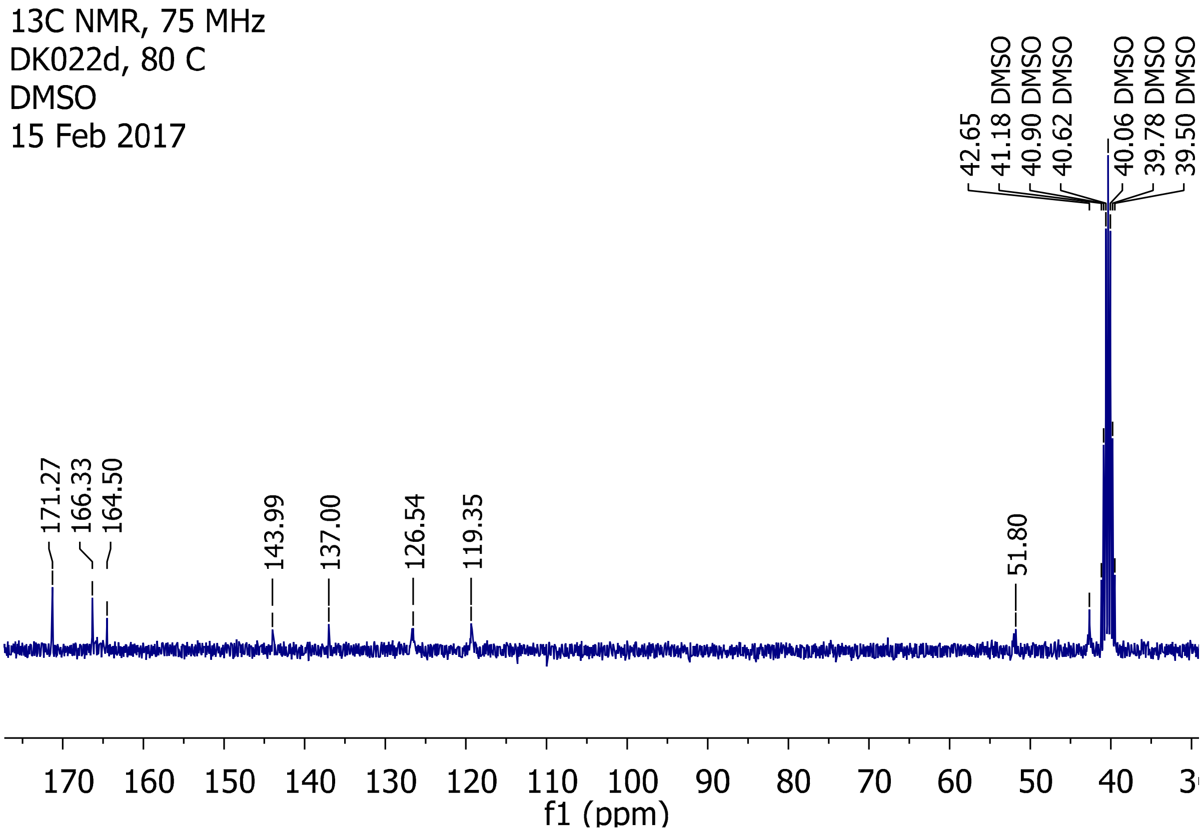


**Figure S2:** $^{1}H$ and $^{13}C$ NMR spectra of dimethyl 2'',2'''-[6'-(4-sulfamoylphenylamino)-1',3',5'-triazine-2',4'-diyl] -bis(azanediyl)diacetate **2**

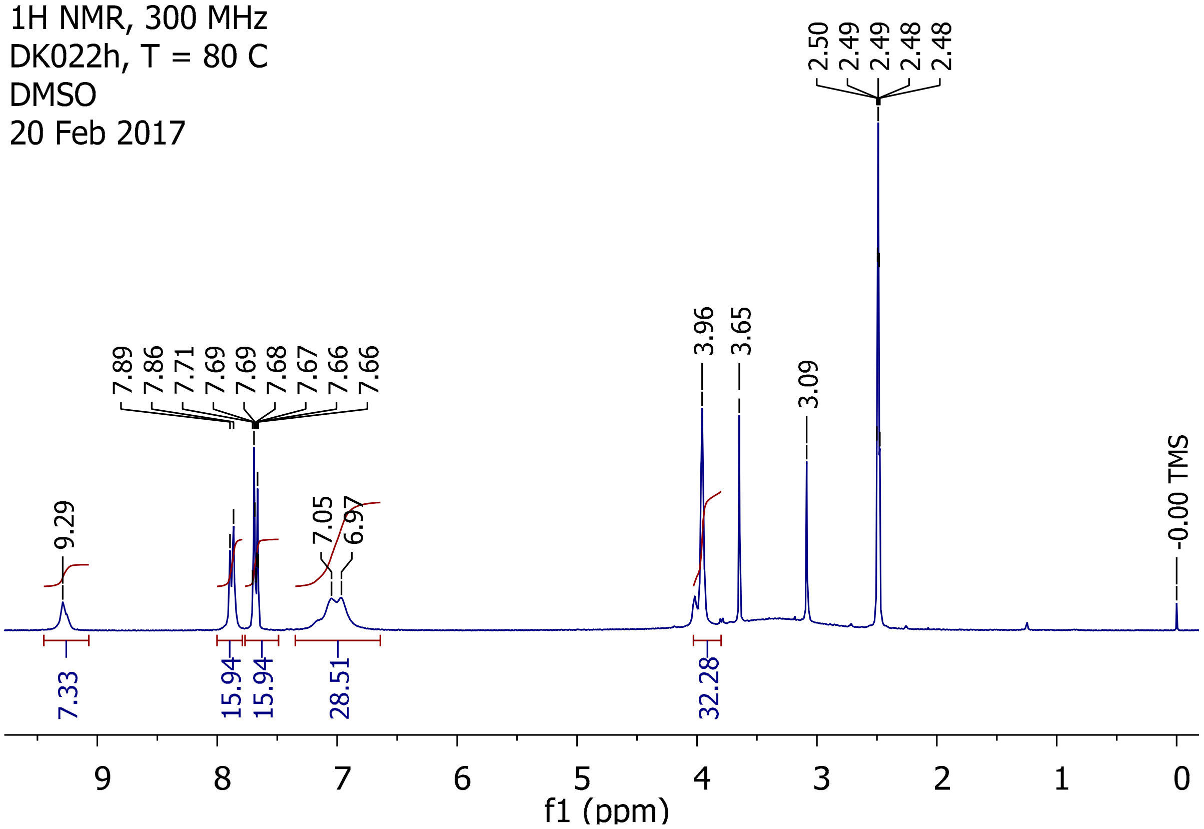


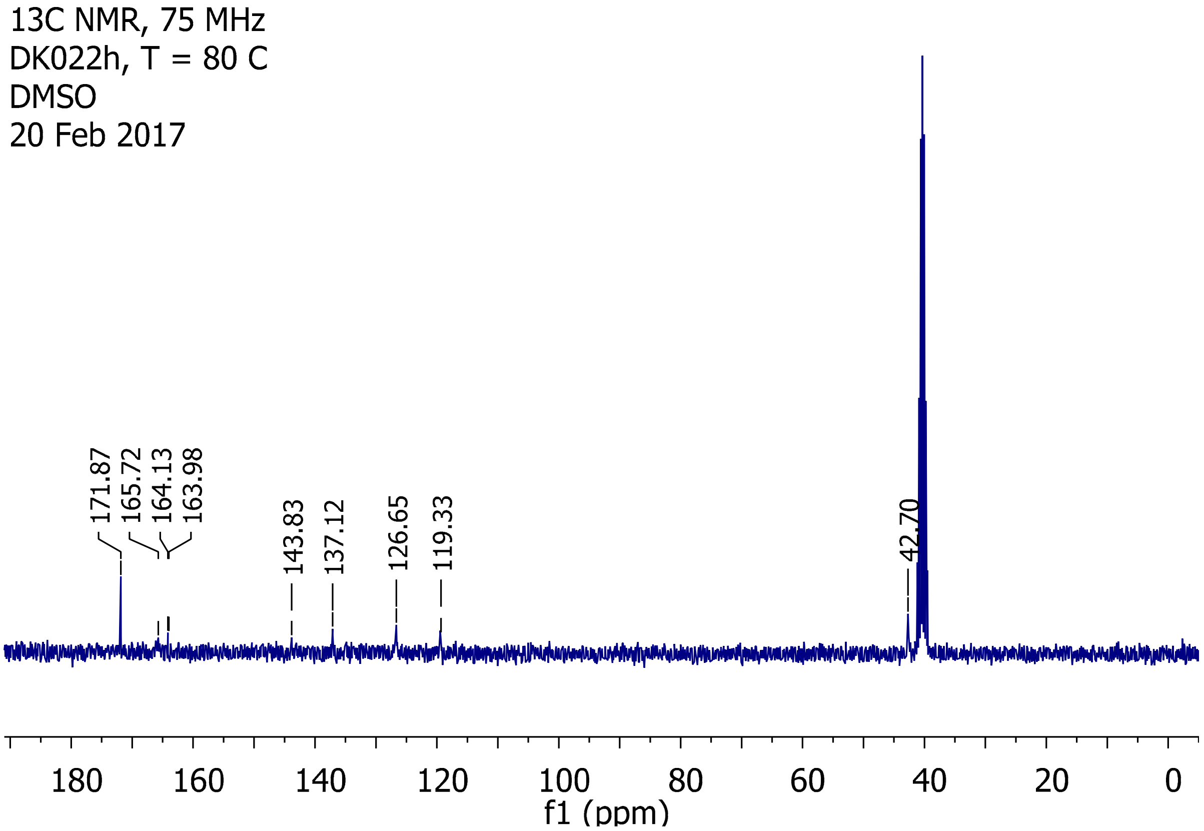


**Figure S3:** $^{1}H$ and $^{13}C$ NMR spectra of 2'',2'''-[6'-(4-sulfamoylphenylamino)-1',3',5'-triazine-2',4'-diyl]-bis(azanediyl)diacetic acid **3** prepared according to the *Scheme 3*

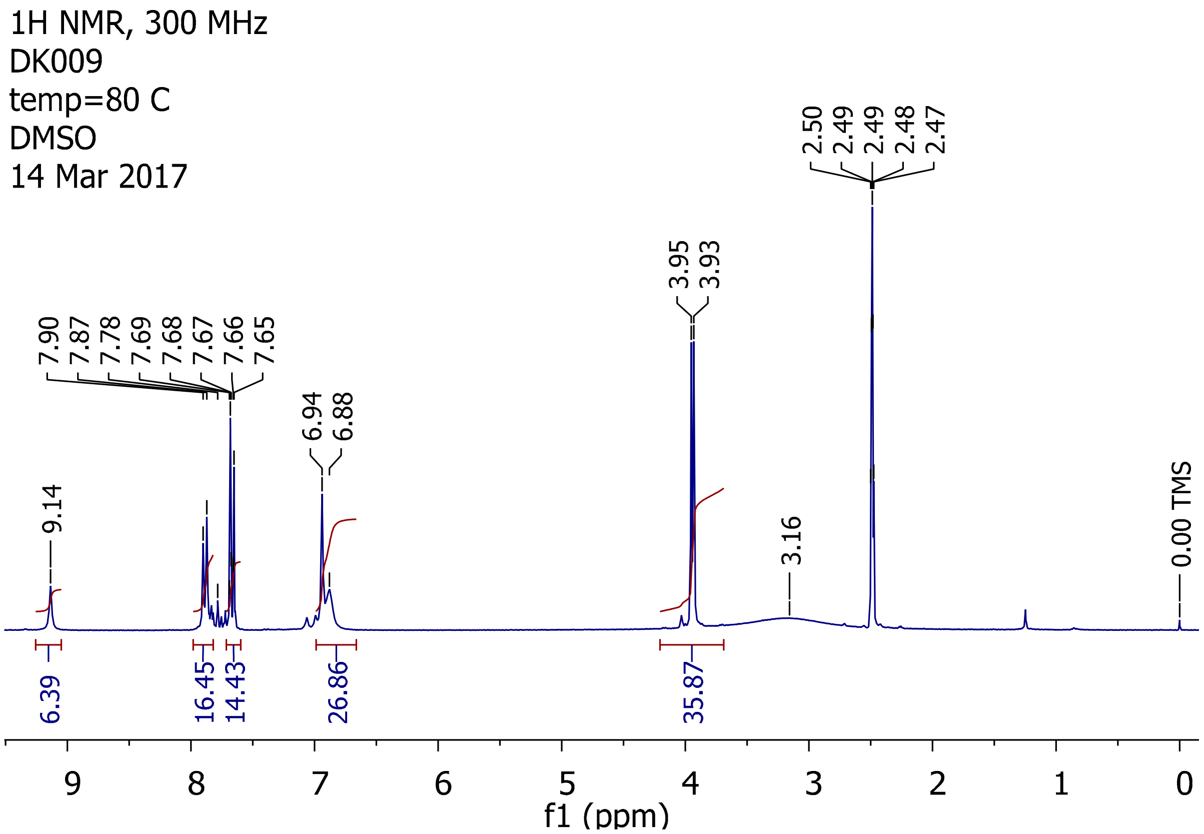


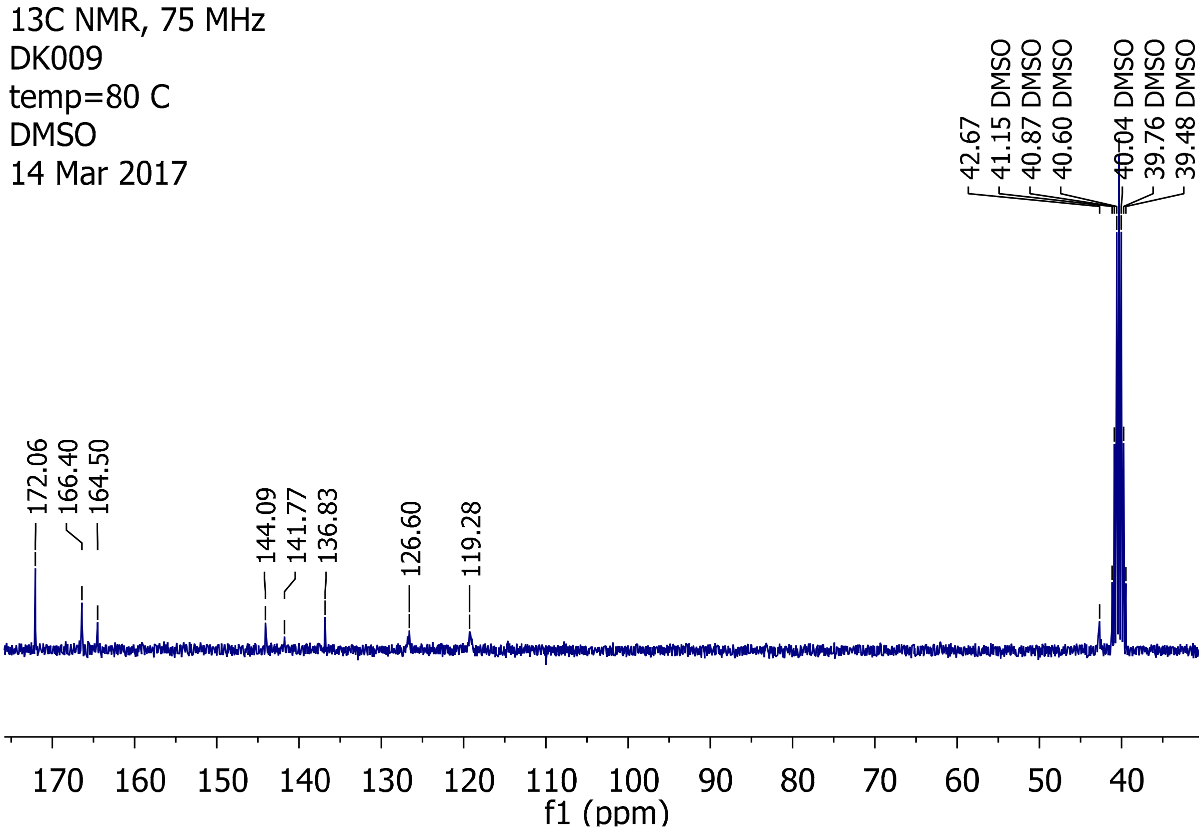


**Figure S4:** $^{1}H$ and $^{13}C$ NMR spectra of 2'',2'''-[6'-(4-sulfamoylphenylamino)-1',3',5'-triazine-2',4'-diyl]-bis(azanediyl)diacetic acid **3** prepared according to the *Scheme 4*

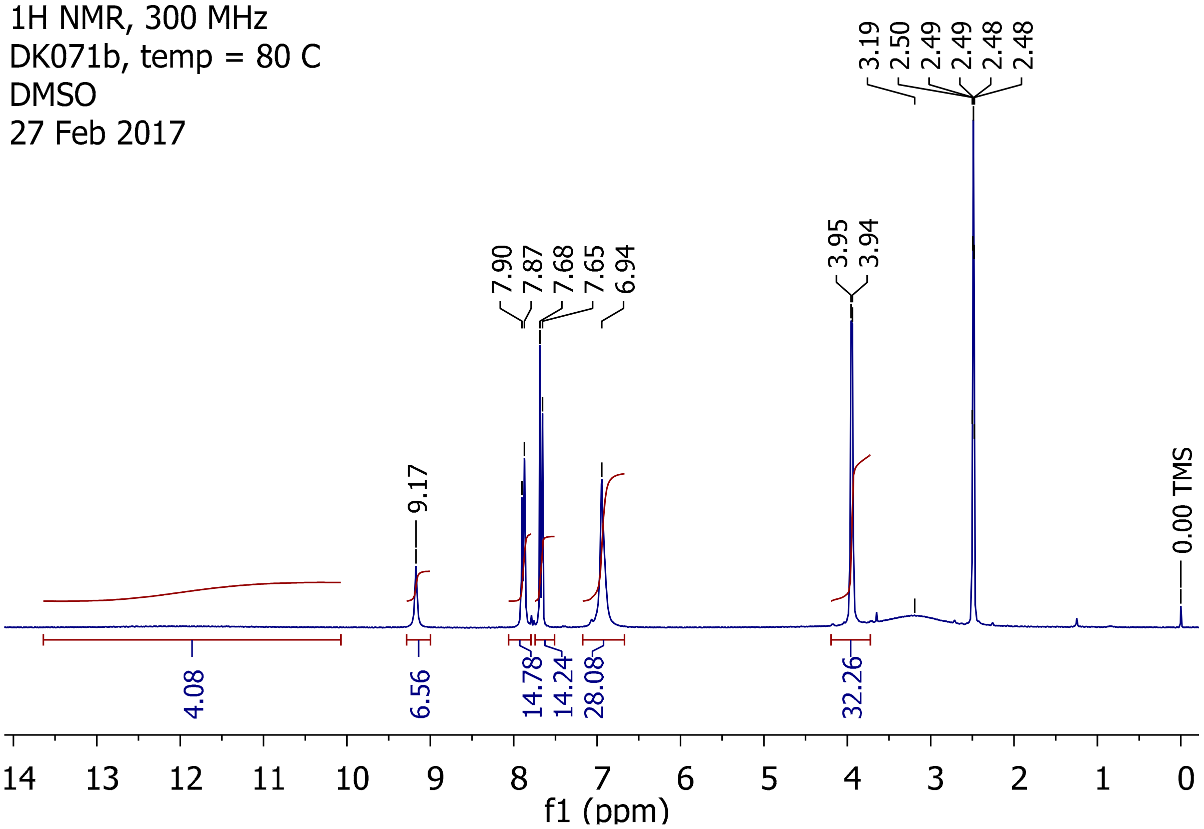


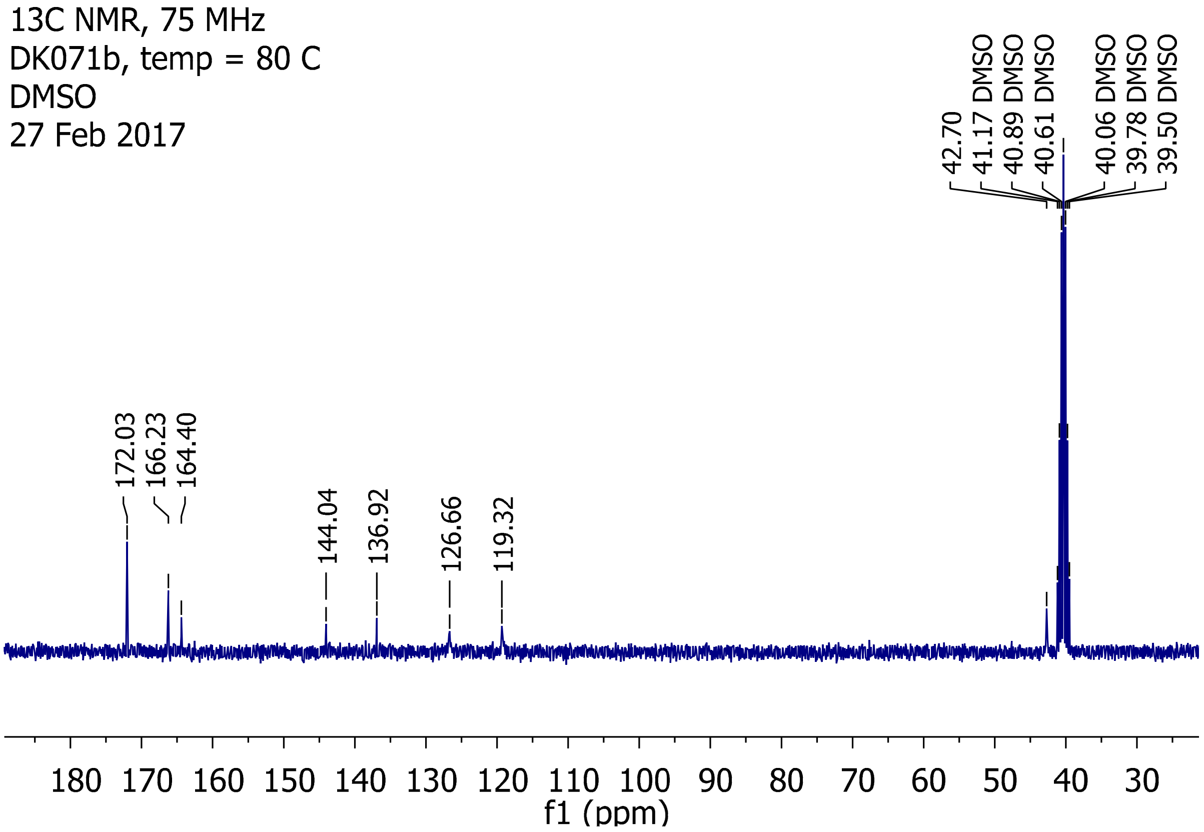


**Figure S5:** $^{1}$H and $^{13}$C NMR spectra of 2'',2'''-[6'-(4-sulfamoylphenylamino)-1',3',5'-triazine-2',4'-diyl]-bis(azanediyl)diacetic acid **3** prepared according to the *Scheme 5*

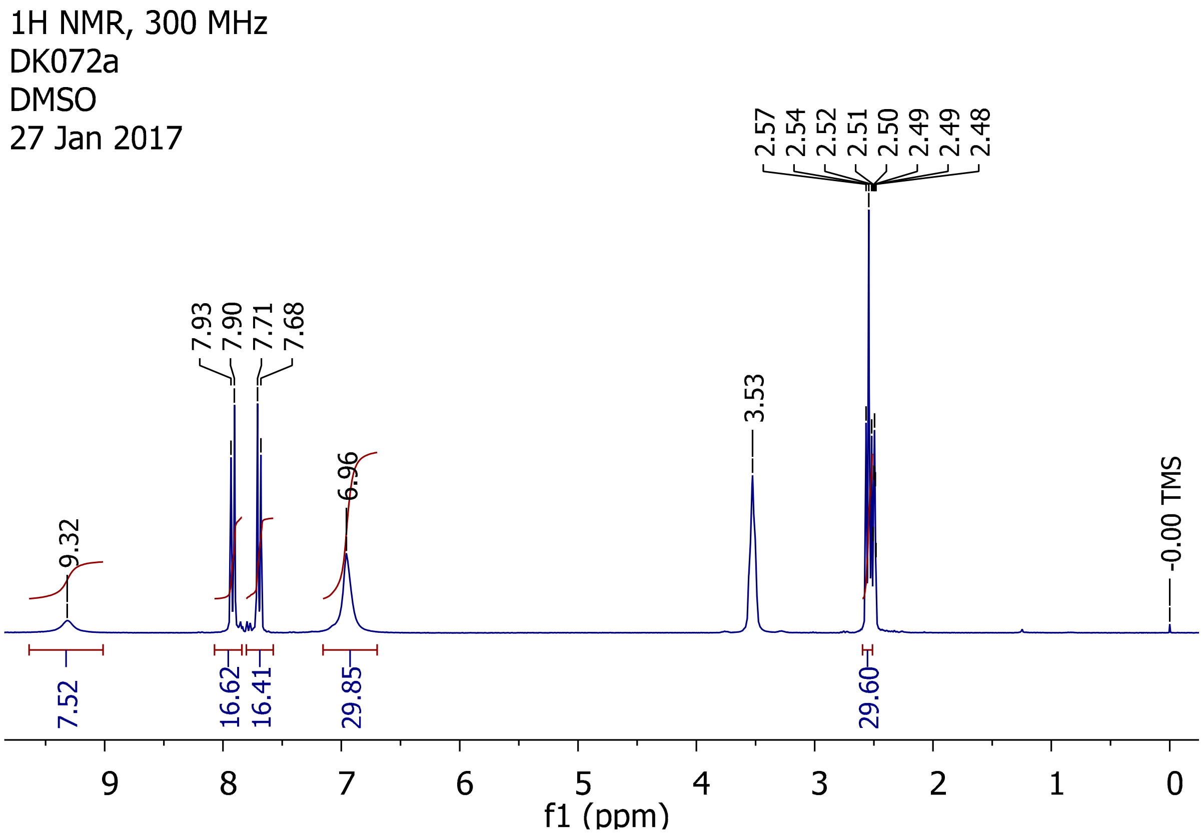


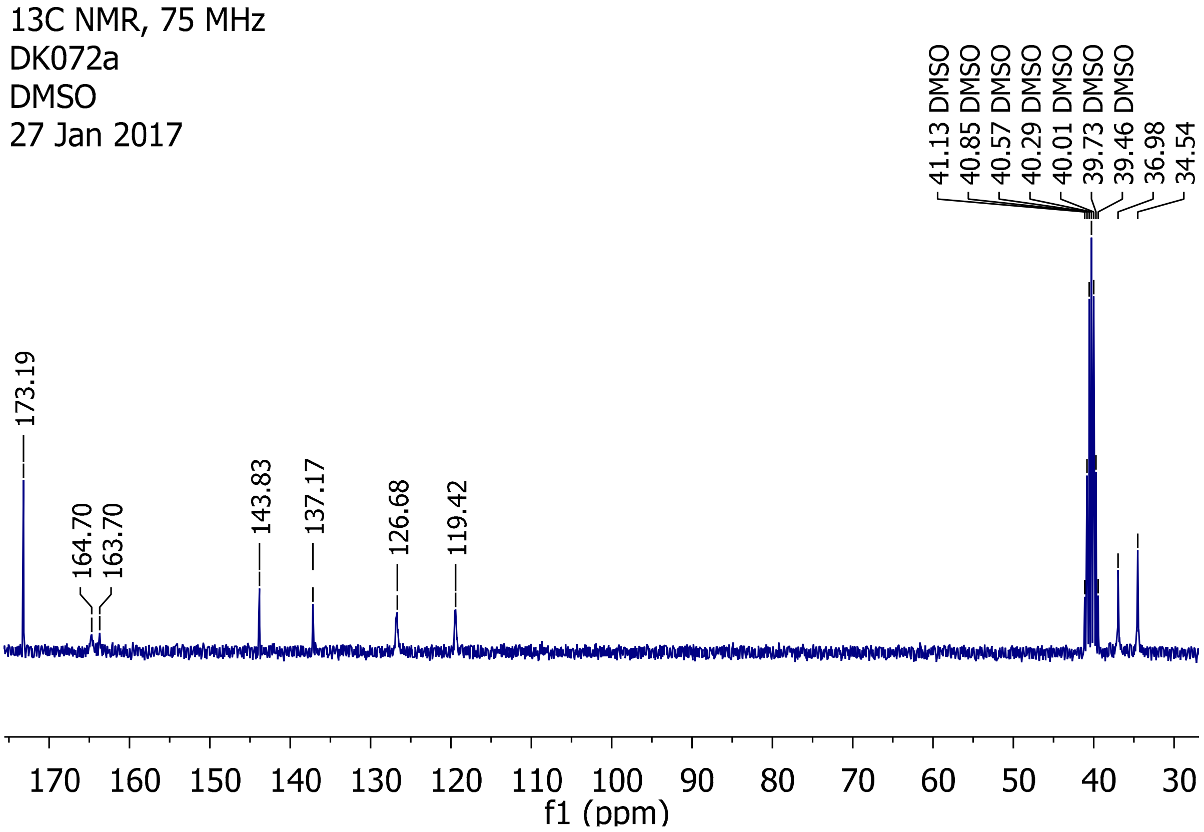


**Figure S6:** $^{1}$H and $^{13}$C NMR spectra of 3'',3'''-[6'-(4-sulfamoylphenylamino)-1',3',5'-triazine-2',4'-diyl]- bis(azanediyl)dipropanoic acid **4**

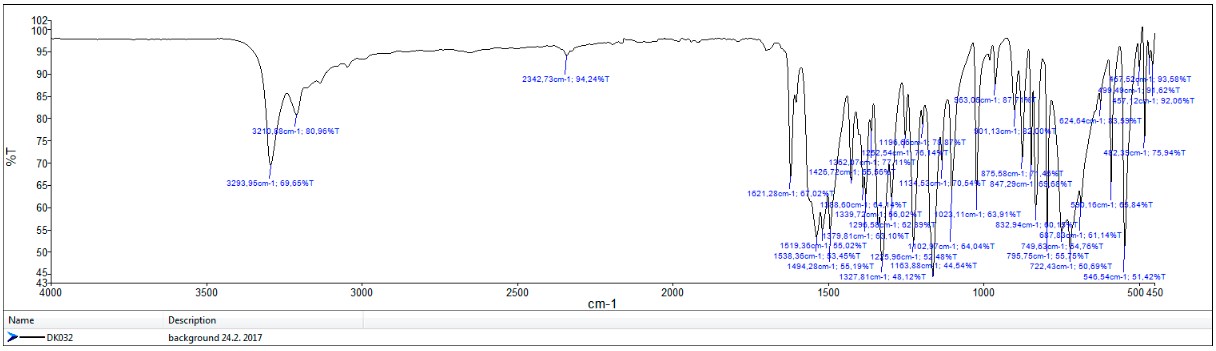


**Figure S7:** IR spectrum of 4-(4',6'-dichloro-1',3',5'-triazin-2'-ylamino)-benzenesulfonamide **1**

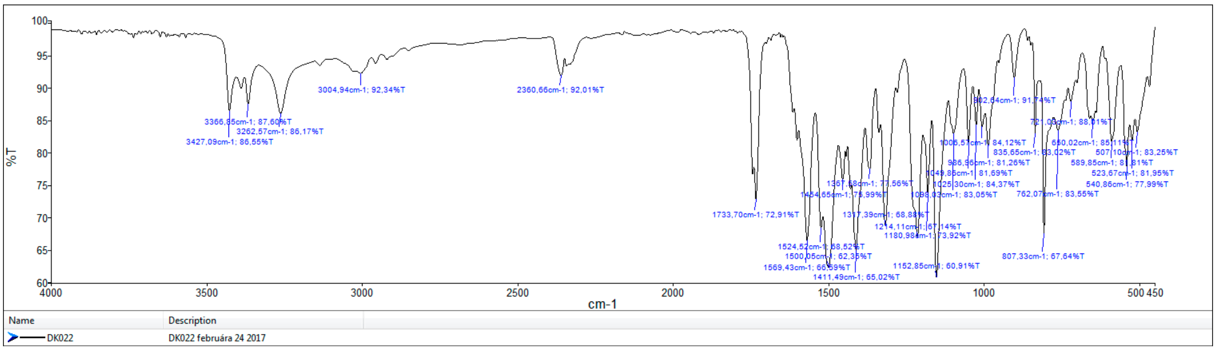


**Figure S8:** IR spectrum of dimethyl 2'',2'''-[6'-(4-sulfamoylphenylamino)-1',3',5'-triazine-2',4'-diyl]-bis(azanediyl) diacetate **2**

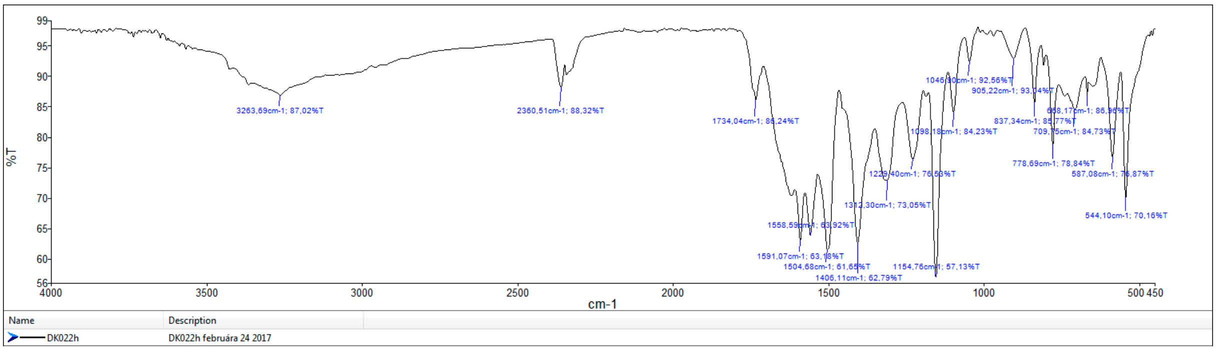


**Figure S9:** IR spectrum of 2'',2'''-[6'-(4-sulfamoylphenylamino)-1',3',5'-triazine-2',4'-diyl]-bis(azanediyl)diacetic acid **3** prepared according to the *Scheme 3*

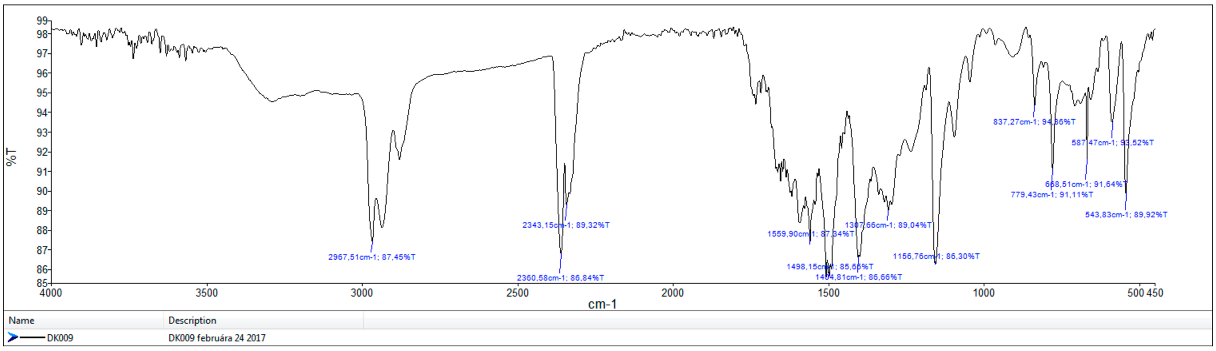


**Figure S10:** IR spectrum of 2'',2'''-[6'-(4-sulfamoylphenylamino)-1',3',5'-triazine-2',4'-diyl]-bis(azanediyl)diacetic acid **3** prepared according to the *Scheme 4*

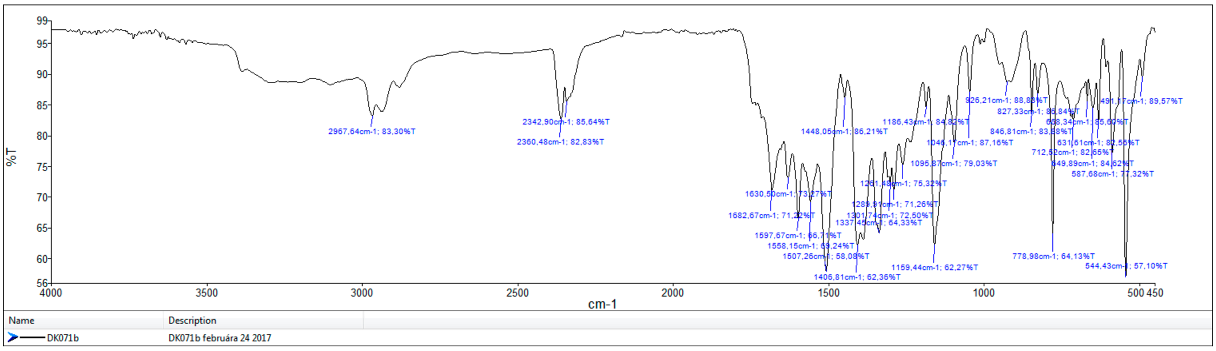


**Figure S11:** IR spectrum of 2'',2'''-[6'-(4-sulfamoylphenylamino)-1',3',5'-triazine-2',4'-diyl]-bis(azanediyl)diacetic acid **3** prepared according to the *Scheme 5*

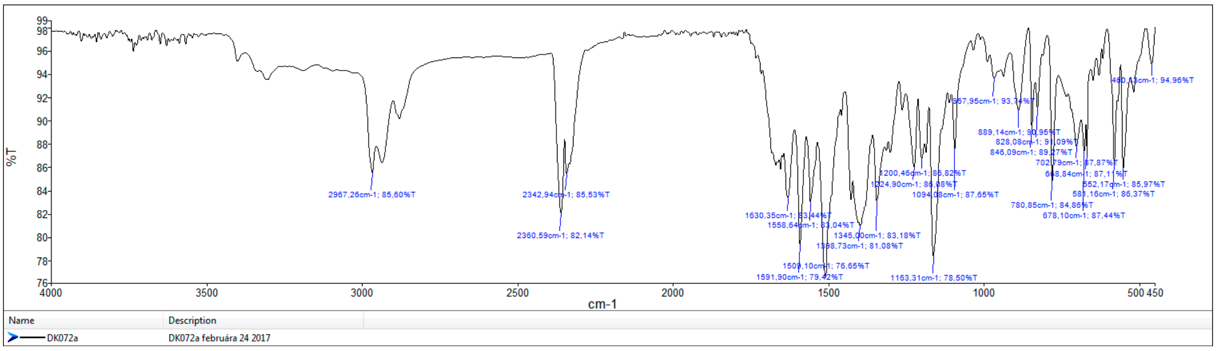

**Figure S12:** IR spectrum of 3'',3'''-[6'-(4-sulfamoylphenylamino)-1',3',5'-triazine-2',4'-diyl]bis(azanediyl) dipropanoic acid **4**

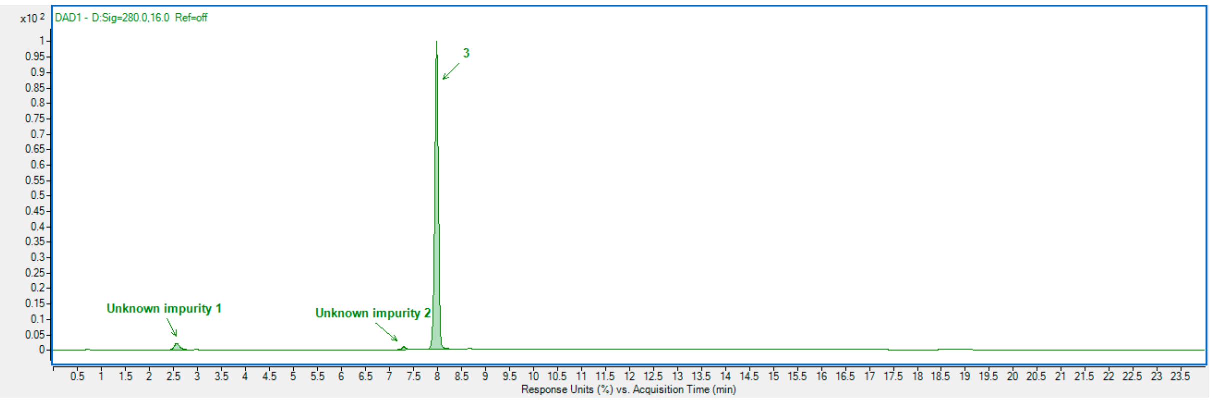


**Figure S13:** HPLC-UV (280 nm) profile of 2'',2'''-[6'-(4-sulfamoylphenylamino)-1',3',5'-triazine-2',4'-diyl]-bis(azanediyl)diacetic acid **3**. Calculated purity of the compound **3** (based on peak areas acquired by HPLC-UV) was 95.51%. Two unknown impurities (3.45% and 1.04%) were detected in the chromatogram. For the analytical conditions see section 3.1.

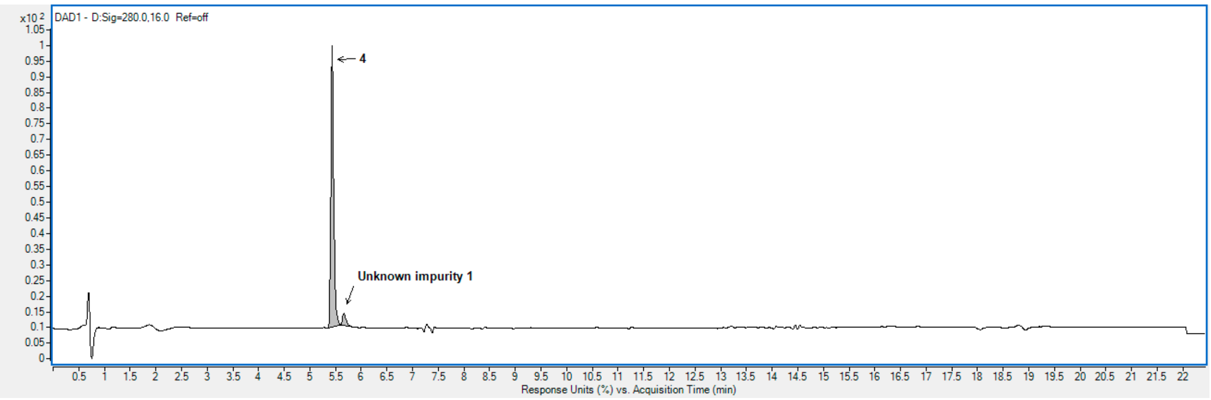


**Figure S14:** HPLC-UV (280 nm) profile of 3'',3'''-[6'-(4-sulfamoylphenylamino)-1',3',5'-triazine-2',4'-diyl]-bis (azanediyl)dipropanoic acid **4**. Calculated purity of the compound **4** (based on peak areas acquired by HPLC-UV) was 94.98%. One unknown impurity (5.02%) was detected in the chromatogram. For the analytical conditions see section 3.1.
